# A multinational phase IIb/III trial of beraprost sodium, an orally active prostacyclin analogue, in patients with primary glomerular disease or nephrosclerosis (CASSIOPEIR trial), rationale and study design

**DOI:** 10.1186/1471-2369-15-153

**Published:** 2014-09-19

**Authors:** Hidetomo Nakamoto, Toshiro Fujita, Hideki Origasa, Masanao Isono, Hajimu Kurumatani, Kiyonobu Okada, Hiroyuki Kanoh, Takashi Kiriyama, Shunsuke Yamada

**Affiliations:** Department of General Internal Medicine, Saitama Medical University, 38 Morohongo, Moroyamamachi, Iruma-gun, Saitama, 350-0495 Japan; Division of Clinical Epigenetics, Research Center for Advanced Science and Technology, The University of Tokyo, 4-6-1 Komaba, Meguro-ku, Tokyo, 153-8904 Japan; Division of Biostatistics and Clinical Epidemiology, Graduate School of Medicine and Pharmaceutical Sciences, The University of Toyama, 2630 Sugitani, Toyama city, 930-0194 Japan; Toray Industries, Inc, Nihonbashi Mitsui Tower, 1-1, Nihonbashi-Muromachi 2-chome, Chuo-ku, Tokyo, 103-8666 Japan; Astellas Pharma Inc, 2-5-1 Nihonbashi-honcho, Chuo-ku, Tokyo, 103-8411 Japan

**Keywords:** Prostacyclin analogue, PGI_2_, Beraprost sodium, Chronic kidney disease, Primary glomerular disease, Nephrosclerosis, Serum creatinine, Renal composite endpoint, CASSIOPEIR, TRK-100STP

## Abstract

**Background:**

Chronic kidney disease (CKD) is public health concern even in Asian countries. TRK-100STP, a sustained release tablet of an orally-active prostacyclin analogue, beraprost sodium, is suggested to suppress worsening of some parameters of renal filtration function, containing in slope of 1/serum creatinine (1/SCr) vs. time in a phase II clinical trial.

**Methods/design:**

We describe the design of the phase IIb/III trial of TRK-100STP, CASSIOPEIR (**C**RF **As**ian **S**tudy w**i**th **O**ral **P**GI_2_ derivative for **E**valuating **I**mprovement of **R**enal function) conducted in approximately 160 centers in China, Hong Kong, Japan, Malaysia, Republic of Korea, Taiwan, and Thailand. A total of 750 patients (n = 250 per group) with primary glomerular disease or nephrosclerosis were planned to be enrolled. Patients were randomized into one of three treatment groups in a double-bind, placebo-controlled manner: TRK-100STP 60 μg b.i.d.; TRK-100STP 120 μg b.i.d.; or placebo. The treatment period is planned to last 2 to 4 years. The primary efficacy endpoint is the renal composite endpoint including doubling of SCr and ESRD (dialysis induction, renal transplantation, or increase in SCr to ≥6.0 mg/dL).

**Discussion:**

This trial targeting CKD patients is designed to (a) demonstrate the superiority of TRK-100STP over placebo using renal composite endpoints, (b) determine the recommended clinical dose, and (c) assess the safety of TRK-100STP in this population and setting.

**Trial registration:**

ClinicalTrials.gov Identifier: NCT01090037.

**Electronic supplementary material:**

The online version of this article (doi:10.1186/1471-2369-15-153) contains supplementary material, which is available to authorized users.

## Background

The number of patients with end-stage renal disease (ESRD) requiring dialysis or renal transplantation has increased globally in recent years. Among the primary diseases that lead to ESRD are the primary and secondary glomerular diseases such as diabetic nephropathy and tubulointerstitial nephritis.

Primary glomerular diseases and nephrosclerosis still comprise a significant proportion of chronic kidney disease (CKD) patients. Primary glomerular diseases are especially important causes for the need for dialysis in Asian countries. They are currently the most frequent causes in China [1] and the second in most other Asian countries [2–4]. Nephrosclerosis is the third frequent cause in most Asian countries and has been increasing [1–5].

The primary care of CKD treatment is to delay renal function deterioration. Current treatments include dietary restriction and antihypertensive drug therapy. Angiotensin-converting enzyme inhibitors (ACEIs) and angiotensin-II receptor blockers (ARBs), which are often administered to both diabetic nephropathy and non-diabetic CKD patients, have been established as the recommended treatment agents for non-diabetic nephropathy patients [6], although either ACEIs or ARBs are insufficient for the prevention of progressive renal disease. Therefore, an additional treatment that is able to significantly delay the progression of CKD is highly coveted.

To meet this unmet medical need, clinical trials have been conducted attempting to delay CKD progression with combination therapies using renin-angiotensin system (RAS) inhibitors. Unfortunately, these trials failed to show an improvement in efficacy with composite renal endpoints while adverse events increase in some of them. Clinical benefits of combination treatments with multiple RAS inhibitors are therefore unproven, at least for diabetic nephropathy [7–10].

Furthermore, a recent trial of Nrf2-activating bardoxolone methyl in diabetic nephropathy patients (BEACON) was prematurely terminated due to safety concerns [11]. A trial of an orally administered activated carbon preparation (AST120: Kremezin®), which is currently used in clinical practice for chronic renal failure in Japan, Republic of Korea and Taiwan, also failed to meet the efficacy endpoints in composite renal endpoint in the large EPPIC randomized controlled trials [12]. The development of a novel CKD treatment strategy is therefore increasingly needed.

Beraprost sodium (BPS) is a stable orally-active prostacyclin (PGI_2_) analogue discovered by Toray Industries, Inc. to overcome instability of prostacyclin *in vivo* [13]. BPS has a novel mechanism for suppressing the progression of CKD. This treatment also targets renal hypoxia, reflecting a growing body of evidence implicating this as a valid therapeutic approach [14]. BPS has been found to be effective in various animal models of renal disorders [15–19]. In addition, BPS immediate-release tablets prevented the decrease in renal blood flow and function in patients with chronic renal failure [20, 21].

Using sustained release tablets of BPS (TRK-100STP), a randomized double-blind placebo-controlled comparative phase II trial aiming to determine the optimal clinical dose was conducted from 2005 to 2008 in Japanese patients with primary glomerular disease or nephrosclerosis as the primary disease [22]. In this trial, serum creatinine (SCr) values were 1.5 to 4.5 for male and 1.3 to 4.0 for female at the initiation of treatment. Randomized patients received oral TRK-100STP 120 μg/day (60 μg b.i.d.), 240 μg/day (120 μg b.i.d.) or placebo for 28 weeks after the 22-week run-in period. The primary endpoint was the difference in slope of 1/SCr vs. time between the run-in period and the treatment period. No statistical difference in the primary endpoint was found between the high-dose (240 μg) group and the placebo group and so a clinical dose recommendation was not determined. Both treatment groups showed better outcomes than the placebo group, with the low-dose (120 μg/day) group showing the highest efficacy. Both active dose groups showed similar effectiveness for additional efficacy endpoints including creatinine clearance, SCr ratio and serum cystatin C, compared to those of the placebo group.

A subgroup analysis was also conducted in the same study among patients with SCr 2.0 mg/dL or higher, whose renal function deterioration was considered irreversible [23]. Deteriorations in all the representative renal filtration functions (i.e. difference in the slope of the 1/SCr vs. time between the run-in period and the treatment period, SCr ratio, serum cystatin C and eGFR) were suppressed in the treatment groups and significant suppressions have been observed in some of these parameters. On the other hand, no clinically relevant safety issues were observed.

Based on these results, it was judged that a hypothesis could be set up to be verified in the phase IIb/III trial. The clinically recommended dose is to be re-evaluated using the same doses as ones in the phase II trial.

This paper details the design and methodology of a phase IIb/III trial (CASSIOPEIR: **C**RF **As**ian **S**tudy w**i**th **O**ral **P**GI_2_ derivative for **E**valuating **I**mprovement of **R**enal function) conceived on the basis of the evidence and findings mentioned above in order to investigate whether TRK-100STP is superior to placebo in treating patients with primary glomerular disease or nephrosclerosis. Evaluating composite renal endpoints, the trial aims also to determine a recommended therapeutic dose of TRK-100STP.

## Methods/design

### Aim of trial

This is a multicenter, randomized, double-blind, placebo-controlled, parallel-group comparative trial targeting CKD patients designed to: (a) demonstrate the superiority of TRK-100STP over placebo using renal composite endpoints, including the extension of the time to the start of dialysis treatment; (b) determine the recommended clinical dose by comparing the low-dose (120 μg/day), high-dose (240 μg/day), and placebo groups; and (c) assess the safety of TRK-100STP in this population and setting.

### Study design

The trial is being conducted in approximately 160 sites, including centers in China, Hong Kong, Japan, Malaysia, Republic of Korea, Taiwan and Thailand. The protocol has been approved by the Ethics Review Committee/Institutional Review Board affiliated with each center. The trial is being conducted in accordance with Good Clinical Practice, and Declaration of Helsinki. All patients provided written informed consent. The trial has been registered on ClinicalTrials.gov, NCT01090037.

**Figure 1:**
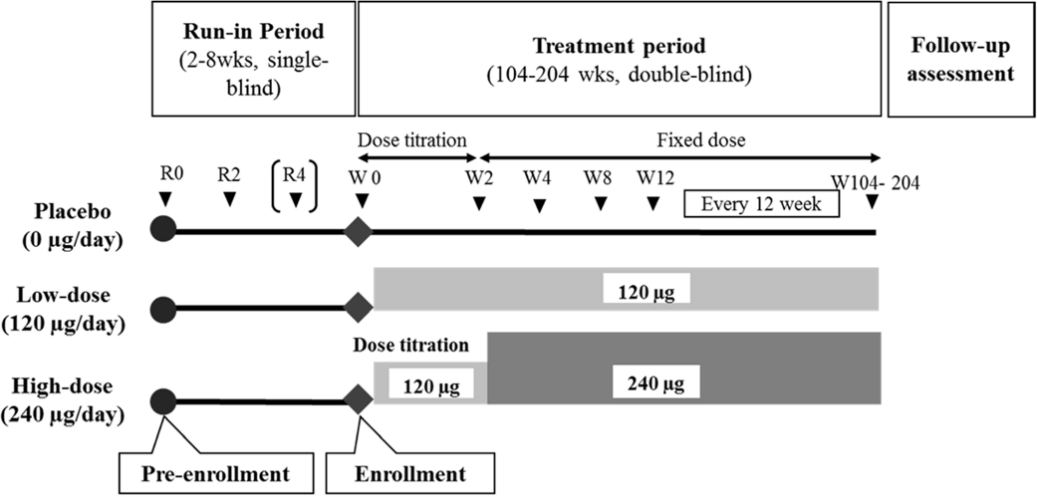
**Schematic diagram of the CRF Asian Study with Oral PGI**_**2**_ **derivative for Evaluating Improvement of Renal function (CASSIOPEIR) study design.**

A total of 750 patients (n = 250 per group) with primary glomerular disease or nephrosclerosis are planned to be randomized. The rationale for setting the sample size at 250 subjects per group is based on “data handling procedure #2: 3-month lag censoring analysis” as follows. In the event of doubling of SCr or increase in SCr to ≥6.0 mg/dL, the 2.5-year event rate estimated from subjects with SCr ≥2.0 mg/dL that were enrolled in the last phase II trial was 48% for the placebo group and 33% for 240 μg group. Therefore, assuming a 2-year-enrollment period, a 2-year follow-up period, and a 35% dropout rate at 2.5 years for each group, the number of subjects required per group is estimated at 237 (with two-sided 5% significance level and 90% statistical power). For all subjects, placebo was orally administered for 2 to 8 weeks, twice a day, during the run-in period, when subjects were blinded although investigators recognized that subjects received placebo (Figure 1). Prohibited and restricted concomitant medications are detailed in Table 1. Subjects who met the inclusion and exclusion criteria (summarized in Table S2; see Additional file 1) after 2-to-8-week run-in period were randomized into one of three treatment groups for the 2-to-4-year treatment period in a double-bind manner: TRK-100STP 120 μg; TRK-100STP 240 μg; or placebo. Subjects randomized in the 240 μg dose group started with a daily dose of 120 μg for 2 weeks and was then increased to 240 μg/day. While the treatment period was planned to last for 2 to 4 years, this may be extended if the incidence of renal composite endpoint is smaller than expected.

**Table 1:** **Prohibited and restricted concomitant medications**

Prohibited Concomitant Medications	The following medications are prohibited for use during the study period.
	(1) Prostaglandin drugs except for ophthalmic solutions and ointments.
	(2) Fluorinated pyrimidine antifungal agents.
Restricted Concomitant Medications	(1) For ACEIs and ARBs, changing the dose or introducing the medication is not permitted from the obtaining of informed consent to the end of the study drug administration. Antihypertensive drugs other than ACEIs or ARBs are permitted to be used.
	(2) From obtaining of informed consent to the end of the study drug administration, changing the dose or introducing the medication is not permitted for pentoxifylline, dilazep hydrochloride, or dipyridamole.
	(3) From obtaining of informed consent to the end of the study drug administration, NSAIDs are not permitted to be taken for more than four consecutive weeks; and during the week prior to a visit, NSAIDs are not permitted to be taken.

### Study endpoints

The primary efficacy endpoint for the trial is the renal composite endpoint (time to first occurrence of one of the following events): 1) Doubling of SCr; defined as at least a two-fold increase in SCr during the treatment period over the baseline value during the run-in period (mean value of the last two measurement results during the run-in period); and 2) ESRD (occurrence of any of the following: introduction of dialysis, renal transplantation, or increase in SCr to ≥6.0 mg/dL). With regards to the doubling of SCr, and the increase in SCr to ≥6.0 mg/dL, a re-confirmation will be performed with blood sample collection and measurement four or more weeks after the initial date on which these endpoints were confirmed, and once reconfirmed, the initial date will be judged as the date on which these renal composite endpoints are reached. The clinical trial and administration of the study drug will be continued for the subject even after the confirmation that SCr has doubled or increased to ≥6.0 mg/dL until introduction of dialysis or renal transplantation is deemed necessary. If the administration of the study drug is prematurely discontinued during the treatment period, the clinical study will be continued as an efficacy follow-up until introduction of dialysis or renal transplantation is judged to be needed.

Secondary endpoints are as follows: 1) Renal composite endpoint or death (from any causes); 2) doubling of SCr; 3) ESRD; 4) introduction of dialysis; 5) renal transplantation; 6) increase in SCr to ≥6.0 mg/dL; 7) slope of 1/SCr vs. time; and 8) eGFR. In addition, the safety endpoints include adverse events, laboratory tests, vital signs and body weight, and 12-lead electrocardiogram (ECG). Also to be evaluated are miscellaneous efficacy endpoints such as “cardiovascular events or renal composite endpoints”.

All SCr values used for analysis of the endpoints are measured at the central clinical laboratory.

### Analysis set

The full analysis set (FAS) will consist of subjects taking at least one dose of study drug for the treatment period. However, subjects meeting the following descriptions are excluded from the FAS:

1. Subjects providing no data after administration of the study drug for the treatment period;
2. Subjects found not to fulfill the inclusion criterion (2) or (3);
3. Subjects found to meet the exclusion criterion (1), (2), or (6).

Subjects who satisfy all of the following criteria out of FAS constitute the per protocol set (PPS), and are:

1. Subjects meeting the inclusion criteria;
2. Subjects not meeting the exclusion criteria;
3. Subjects providing three or more SCr measurement data points obtained at and after Week 4 of the treatment period (including at least one data point taken at Day 70 of the treatment period or later);
4. Subjects whose drug compliance rate is two-thirds or higher during the administration period of the study drug for the treatment period.

The safety analysis set (SAF) will include subjects taking at least one dose of the study drug for the treatment period.

The pharmacokinetics analysis set (PKAS) is defined by subjects fulfilling all of the following criteria:

1. Subjects taking at least one dose of the study drug for the treatment period;
2. Subjects providing at least one data point (sample) for plasma BPS concentration determination for which information on the date and time of blood collection and medication before the collection is available.

### Handling of data

For efficacy assessment, the following three types of data handling processes have been established:

1. Data Handling Procedure #1
  All data will be adopted for analysis that is obtained after the start of the administration of the study drug for the treatment period (Intension-To-Treat (ITT) analysis).
2. Data Handling Procedure #2
  For subjects who discontinue the administration of the study drug for the treatment period, their data obtained after 3 months following the discontinuation will not be used, and data collection for them is censored at 3 months from the discontinuation or at completion of the study, whichever comes first (3-month lag censoring analysis).
3. Data Handling Procedure #3
  Subjects that take a prohibited concomitant medication will be censored at the time of the medication. Subjects will be censored at any time when they stop taking the study drug for the treatment period, for whatever reason.

### Analysis of efficacy

The primary population for efficacy analysis will be the FAS, and the data will be processed according to the Data Handling Procedure #1. Similar analyses will be conducted according to the Data Handling Procedure #2 in terms of FAS and according to the Data Handling Procedure #3 in terms of PPS. Statistical testing will use two-sided 5% significance level and calculate two-sided 95% confidence interval, unless otherwise specified.

### Analysis of safety

Vital signs, adverse events, laboratory tests, body weight, 12-lead electrocardiogram (ECG) and pregnancy test will be performed for the purpose of safety evaluation.

### Analysis of pharmacokinetics

Analysis of pharmacokinetics will be conducted using PKAS. During the treatment period the participants will make five visits prior to the study drug administration in the morning for a blood test. They will make another six visits after taking the morning dose to have blood samples collected. The time from the drug administration will be recorded, and the serum BPS level is to be determined. The population pharmacokinetics analysis will be conducted in addition to the pharmacokinetics analysis.

### Statistical evaluation

For the analysis of efficacy, the renal composite endpoint will be treated as an event and this evaluation will be conducted in the following steps. Step 1: Based on Cox’s proportional hazard model with the region as a covariate, contrast test will be performed on the comparison coefficient (1, -1/2, -1/2) for the placebo group, 120 μg group, and 240 μg group at two-sided 5% significance level. Step 2: If a statistical significance was observed in Step 1, pair-wise comparison between the placebo and the 120 μg groups and between the placebo group and the 240 μg group will be performed at a two-sided 5% significance level using Cox’s proportional hazard model with the region as a covariate.

### Endpoint judgment committee

Prior to the start of the study, an Endpoint Judgment Committee (EJC) was established. The committee is comprised of three committee members who are not involved directly in the study. The committee will examine, particularly, the validity of dialysis introduction, renal transplantation and cardiovascular events, among the efficacy endpoints in each institution.

### Data and safety monitoring board

Prior to the start of the study, the Data and Safety Monitoring Board (DSMB) was established. The committee is comprised of three committee members who are not involved directly in the study. The safety evaluation committee assesses safety data including serious adverse events.

## Results

The first patient was enrolled in this trial in June 8, 2010. A total of 1105 subjects were enrolled in run-in period. From July 8, 2010 through April 26, 2012 a total of 892 subjects underwent randomization (200 in China, 13 in Hong Kong, 339 in Japan, 46 in Malaysia, 135 in Republic of Korea, 108 in Taiwan and 51 in Thailand).

The EJC and DSMB have been conducted 2 and 3 times to date, respectively and have concluded that there is no concern regarding the continuation of this trial.

## Discussion

This is a multicenter, randomized, double-blind, placebo-controlled, parallel-group comparative trial targeting CKD patients designed to, (a) demonstrate the superiority of TRK-100STP over placebo using renal composite endpoints, (b) determine the recommended clinical dose, which was not established following the respective phase II trial, and (c) assess the safety of TRK-100STP in this population and setting.

Prostacyclin, which was discovered in 1976, was expected to be a key advance for novel pharmacotherapy for various cardiovascular diseases [24]. However, prostacyclin proved to be an unstable chemical substance having a short elimination half-life in blood plasma. The clinical use of prostacyclin was consequently limited to serious cases of pulmonary arterial hypertension (PAH) for which 24-hour continuous infusion is indicated [25]. BPS is a stable orally-active prostacyclin analogue and its immediate-release tablet obtained manufacturing approval for the treatment of chronic arterial occlusive diseases from the Japanese regulatory agency in January 1992 (brand names: Dornar® and Procylin®). An additional indication for primary pulmonary hypertension was approved in September 1999. Besides Japan, BPS immediate-release tablets were launched in other Asian markets including China, Indonesia, Republic of Korea, Thailand and The Philippines, between 1997 and 2006.

TRK-100STP, a hydrogel-forming matrix preparation, provides a sustained-release preparation of BPS [26]. In October 2007, TRK-100STP was given marketing approval in Japan for the treatment of PAH with a maximum daily dose of 360 μg (180 μg b.i.d.) (Brand names: Careload® and Berasus®). The maximum daily clinical dose of TRK-100STP has doubled those of the immediate-release tablet for the indication of pulmonary hypertension in Japan.

BPS has the same pharmacological actions as those of natural prostacyclin, including anti-platelet and vasodilating effects [27], as well as being able to protect vascular endothelial cells [28] and inhibit inflammatory cytokines production including MCP-1 [17].

The importance of tubulointerstitial hypoxia has recently been highlighted as the final common pathway of CKD [29], a notion supported by an increasing body of evidence from animal models [14, 30, 31] and clinical study [32]. Renal blood flow is reduced when renal glomeruli are damaged by pathological factors such as inflammation and hypertension. Lack of oxygen, particularly in the tubulointerstitium of the outer-medullary region which is susceptible to hypoxia, triggers further damage to renal glomeruli which causes renal function to progressively decline [33]. It has been demonstrated, in the glomerular nephritis rat model, that the beneficial effects of BPS are attributed, at least in part, to the amelioration of tubulointerstitial hypoxia [19].

Prostacyclin is a vasodilator which, at a high dose, diminishes blood pressure or brings about increased heart rate (HR) as compensation in several animal species [27]. However, neither increased HR nor lower blood pressure is observed at the clinical dose up to 180 μg b.i.d. in patients with PAH [34]. Additionally, the phase II trial of CKD patients demonstrated that TRK-100STP up to 120 μg b.i.d. does not affect either blood pressure or urinary protein. Thus the effect of BPS to suppress the reduction of renal function is not related to blood pressure lowering or to reducing urinary protein levels. This suggests that the mechanism of beneficial effect of BPS for renal function is different from that of ACEIs/ARBs.

Other features of this trial are as follows:

1. Treatment with TRK-100STP is employed, in principle, as an add-on to existing treatments. No limitation was set against concomitant ACEIs/ARBs use, although dose alternation and newly initiated prescription is restricted. Meanwhile recent evidence from large-scale clinical studies indicates that combination therapy with RAS inhibitors is not effective and also increases certain safety concerns in some of them. The add-on approach was therefore chosen in this trial, based on the observation that a new approach is required beyond RAS inhibitor-based combination therapy.
2. This trial utilizes carefully defined eligibility criteria that allows for sufficient numbers of events while excluding slow-progressing patients. This is important because the pace of renal function deterioration varies widely among patients, especially among non-diabetic CKD patients where disease progression is sometimes very slow. Enrollment is limited to patients with SCr of 2 mg/dL or higher where an irreversible decrease in renal function is reported [23]. Clinical benefits were more clearly apparent in such patients in the previous phase II trial [22]. Participants must also have experienced progression in renal function deterioration, assessed by the slope of 1/SCr vs. time, within one year before the start of the study. These aspects of recruitment may subsequently help us define the target population likely to benefit most from intervention.
3. The necessity of introduction of dialyses or kidney transplants were determined by the principal investigator based on medical need assessment. All endpoints including the dates of introduction of dialyses or kidney transplants, as well as cardiovascular event occurrence, were validated by an EJC whose members are third-party physicians.
4. Patients with diabetes at pre-enrollment were excluded. The findings of several animal model studies have indicated that BPS may be effective against diabetic nephropathy [35–37]. Researchers have also reported that BPS was effective in patients with microalbuminuria [38]. However, there are several methodological difficulties in covering both diabetic and non-diabetic CKD in a single study protocol. In addition, considering there is a great unmet need for non-diabetic nephropathy treatment in Asia, we decided to prioritize and focus on non-diabetic CKD, wherein renal disorders are not modified by diabetes. As it is difficult in practice to definitively distinguish between patients with diabetic nephropathy and non-diabetic CKD patients with diabetes, all patients with diabetes at pre-enrollment are excluded.

In the original trial design, handling of primary data was stipulated as “3-month lag censoring analysis”. However, Japanese regulatory authority, Pharmaceuticals and Medical Devices Agency (PMDA), advised that ITT analysis should be primary analysis. The priority order of data handling was therefore amended based on the PMDA’s advice. Meanwhile, the sample size estimation and rationale for setting the sample size in “study design”, which was designed based on 3-month lag censoring analysis, was not amended.

## Conclusion

This trial evaluates the efficacy and safety of TRK-100STP in the treatment of patients with primary glomerular disease or nephrosclerosis using renal composite endpoints, and also aims to establish a recommended dose of TRK-100STP to optimize clinical outcomes.

### Endpoint judgment committee members

Akira Hishida (Chair), Enyu Imai, Masato Nakamura

### Data and safety monitoring board members

Akira Saito (Chair), Masaki Kobayashi, Naoki Kashihara

### Ethical approval

This trial has been approved by competent regulatory authorities of China, Hong Kong, Japan, Malaysia, Republic of Korea, Taiwan and Thailand. In accordance with the laws of each country, the trial has been conducted after obtaining documented approval from Institutional Review Board/Independent Ethics Committee in each site according to ICH-GCP guidelines.

## Electronic supplementary material

Additional file 1: Table S2: Study inclusion and exclusion criteria. (DOCX 17 KB)
