# Supplementary material for: A multinational phase IIb/III trial of beraprost sodium, an orally active prostacyclin analogue, in patients with primary glomerular disease or nephrosclerosis (CASSIOPEIR trial), rationale and study design
Source: BMC Nephrol. 2014 Sep 19;15:153. doi: 10.1186/1471-2369-15-153 (PMC4181382; doi:10.1186/1471-2369-15-153)
Supplement: Supplementary file 1 — Additional file 1: Table S2: Study inclusion and exclusion criteria. (DOCX 17 KB) [file 12882_2014_843_MOESM1_ESM.docx]

**Additional file 1**

**Table S2.** Study inclusion and exclusion criteria

| **Inclusion criteria** |
| --- |
| Patients enrolled in the study must meet *all* of the following criteria:  *[At pre-enrollment]*   1. Of the legal age (country-specific), and is <76 years old at the time of informed consent. 2. Has provided written informed consent. 3. CKD patient with primary glomerular disease or nephrosclerosis as the primary disease. 4. Patients with progressive CKD presenting with a slope of ≤ -0.005 dL/mg over 4 weeks for the least-squares regression line plotted with at least three 1/SCr data points* . These data should include all SCr values measured within 1 year before submission of informed consent. In addition, these data should contain at least one measurement result obtained 6 or more months before the submission of the informed consent. 5. Patients whose SCr measurements used in inclusion criteria (4) are >0.5 times and <2.0 times the SCr measured at the last point before the date of informed consent.   *[At enrollment to the treatment period]*  (6) SCr measurement at the last 2 points during the run-in period are within 2.00-4.50 mg/dL.  *: “≤ -0.005 dL/mg over 4 weeks in the slope of 1/SCr vs.time” is equivalent to “≤ -2.5 mL/min/1.73m^2^/year in eGFR.” |
| **Exclusion criteria** |
| If *any* of the following criteria are met, they will not be enrolled in the study:  *[At pre-enrollment]*   1. Glomerular disease is secondary to diabetic nephropathy, lupus nephritis, ANCA-related nephritis, renal amyloidosis, hepatitis virus-associated nephropathy, Wegener's granulomatosis, purpuric nephritis, hereditary nephritis (Alport's syndrome), Fabry's disease, benign familial hematuria (thin glomerular basement membrane syndrome), kidney hypoplasia, etc. 2. CKD caused by pyelonephritis, interstitial/tubular nephritis, gouty kidney, polycystic kidney disease, or nephroureterolithiasis. 3. Has been administered an oral/injectable steroid agent, newly or increase in dose for treatment of a kidney disease during the 1 year prior to the date of informed consent. 4. Has taken NSAIDs during the 1 week prior to the date of informed consent. However, topical medications (other than suppositories), and aspirin medications (up to 324 mg/day) are acceptable. 5. Diabetes mellitus, nephrotic syndrome or renal-artery stenosis. 6. A history of undergoing renal transplantation, hemodialysis or peritoneal dialysis within a year prior to the date of informed consent; or the patient who is planned for renal transplantation, hemodialysis, or peritoneal dialysis within 24 weeks following the date of informed consent. 7. Patients with malignant hypertension or uncontrolled hypertension (with a sitting systolic blood pressure of ≥ 150 mmHg, or a sitting diastolic blood pressure of ≥ 95 mmHg, at three latest measurement points before the date of informed consent). 8. Serious hepatic disease, blood dyscrasia, respiratory disease, gastrointestinal disease, heart disorder, cerebellar or cerebral disorders. 9. Taken BPS during the one year prior to the date of informed consent. 10. Taken TRK-100STP during the phase II clinical trial [chronic renal failure (primary glomerular disease/nephrosclerosis)].   *[At enrollment to the treatment period]*  At the time of enrollment, it will be confirmed that none of the exclusion criteria in the pre-enrollment (1) to (10) applies to the patient. However, at the time of enrollment, exclusion criteria (7) in the pre-enrollment will be replaced as follows:  The patient's condition is complicated by malignant hypertension or uncontrolled hypertension (with a sitting systolic blood pressure of ≥150 mmHg, or a sitting diastolic blood pressure of ≥ 95 mmHg, in all of the measurements taken during the run-in period). |
